# Regenerative Potential of Autologously Processed White Adipose Tissue for Peripheral Nerve Regeneration: Evaluation of Growth Factor Profiles and Electrical Stimulation

**DOI:** 10.3390/cells15141250

**Published:** 2026-07-10

**Authors:** Tobias Egger, Andreas Eigenberger, Oliver Felthaus, Marc Ruewe, Luis Sturz, Christian Festbaum, Dmytro Oliinyk, Andreas Siegmund, Tom Schimanski, Katharina Rosengarth, Daniel Deuter, Philipp Kreiner, Lukas Prantl, Silvan M. Eisenmann

**Affiliations:** 1 Department of Plastic, Hand and Reconstructive Surgery, University Hospital Regensburg, 93042 Regensburg, Germany; 2 Department of Neurosurgery, University Hospital Regensburg, 93042 Regensburg, Germany; 3 Department of General Surgery, University Hospital Regensburg, 93042 Regensburg, Germany

**Keywords:** adipose-tissue-derived stem cells, nerve regeneration, neurotrophic factors, electrical stimulation

## Abstract

**Highlights:**

**What are the main findings?**

- Standardized mechanical intersyringe processing of human lipoaspirate indicated increased relative expression levels of key neurogenic and angiogenic growth factors in CELT^PLUS^ compared to CELT processing.
- Lipoaspirate displayed time-dependent release kinetics of regenerative factors; furthermore, external electrical stimulation was associated with an increased release of NGF.

**What are the implications of the main findings?**

- Processed lipoaspirate preparations exhibit increased expression of growth factors relevant to regeneration after peripheral nerve injury.
- The findings suggest that both CELT^PLUS^ processing and electrical stimulation are associated with a shift toward a more pro-regenerative expression pattern.

**Abstract:**

Peripheral nerve injuries (PNI) present a major clinical and socioeconomic challenge due to limited regenerative capacity. Adipose-derived stem cells (ADSCs) within the stromal vascular fraction (SVF) of white adipose tissue offer a promising autologous source for regenerative support. This study evaluated the impact of mechanical processing CELT (Cell-Enriched Lipotransfer) and CELT^PLUS^ and electrical stimulation on the regenerative secretome of human lipoaspirates. qPCR analysis revealed that CELT^PLUS^ processing, which incorporates mechanical intersyringe shifting, significantly doubled the gene expression of nerve growth factor (*NGF*) (*p* = 0.015), vascular endothelial growth factor (*VEGF*) (*p* = 0.02), and brain-derived neurotrophic factor (*BDNF*) (*p* = 0.04) compared to CELT-processed lipoaspirate. Protein analysis via ELISA confirmed a time-dependent secretion of NGF and VEGF over 96 h. Furthermore, 24 h electrical stimulation (2 V) significantly enhanced NGF protein release (*p* < 0.001). These findings demonstrate that standardized mechanical processing effectively enriches regenerative cell populations and amplifies their neurogenic and angiogenic potential. The additional modulation of growth factor secretion via electrical stimulation highlights the potential of processed adipose tissue as a functional, autologous transplant for enhanced nerve reconstruction.

## 1. Introduction

The restoration of neural function following peripheral nerve injury (PNI) is a protracted process, often spanning months to years [1,2]. Beyond individual impairment, PNIs impose a substantial socioeconomic burden due to long-term disability and loss of productivity [3,4,5]. Consequently, research is focused on therapeutic strategies to accelerate regeneration and enhance qualitative outcomes [6,7]. In traumatic cases, prompt surgical intervention is mandatory, with the technique dictated by the defect length. While primary epineural or perineural suturing is preferred for tension-free repairs, larger defects exceeding several centimeters necessitate autologous nerve grafts [7,8]. Despite being the gold standard, autologous nerve transplantation is limited by donor-site morbidity, restricted graft availability, and dimensional mismatch, while direct suturing under tension risks neuroma formation [9]. As alternatives, allografts carry the risk of immune rejection [10]. To overcome these constraints, nerve guide conduits (NGCs), which provide a structural scaffold for axonal guidance, have been developed [11,12]. To augment the efficacy of passive scaffolds, recent experimental models have functionalized NGCs with growth factors to achieve controlled, sustained release. Given their pivotal role in nerve regeneration, the use of NGCs with growth factors has emerged as a promising strategy to restore motor and sensory functions following PNI [13,14,15,16,17,18].

**Figure 1:**
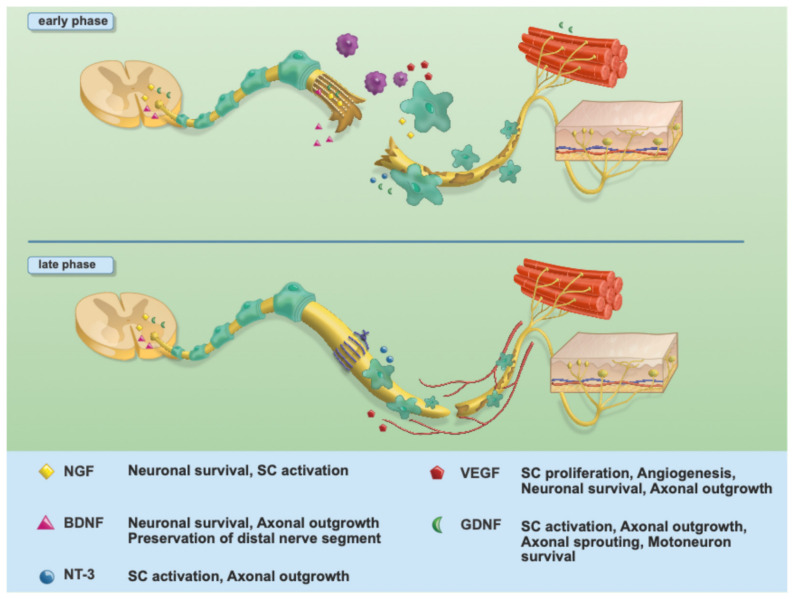
Illustration of the temporal dynamics of neurotrophic factors secreted by the stromal vascular fraction (SVF) during early and late phases of peripheral nerve regeneration. Following primary surgical repair, the endogenous upregulation of these factors can be synergistically enhanced by the external application of lipoaspirate-derived substrates to facilitate the restoration of motor and sensory function. Violet cells indicate activated SCs, while green cells represent myelinating SCs. Skin and muscle tissue represent the nerve target. (NGF: nerve growth factor; VEGF: vascular endothelial growth factor; BDNF: brain-derived neurotrophic factor; NT-3: neurotrophin-3; GDNF: glial-cell-derived neurotrophic factor; SC: Schwann cell).

However, as bioactive peptide factors, these molecules are susceptible to rapid proteolytic degradation and short half-lives in vivo [19]. While NGC coatings can be engineered to facilitate controlled, sustained release, these conduits remain exogenous material, potentially impeding the regenerative trajectory. To circumvent these limitations, attention has shifted toward the targeted application of endogenous, secreting cells directly at the lesion site [20]. White adipose tissue is a heterogeneous tissue that extends beyond mature adipocytes. In addition to immune and endothelial cells, the Stromal Vascular Fraction (SVF) is of particular significance to the present work. A key subpopulation within the SVF consists of Adipose-derived stem cells (ADSCs) [21,22]. ADSCs have emerged as a prime candidate due to their potent secretory profile and ease of high-yield harvest via minimally invasive liposuction [23,24]. It has been demonstrated that ADSCs can secrete a specific profile of growth factors that are essential for the coordination of peripheral nerve regeneration. Furthermore, as mesenchymal progenitors, ADSCs exhibit remarkable multilineage differentiation potential, including neural and endothelial differentiation [25,26,27]. By modifying the lipoaspirate, a significant enrichment of ADSCs is achieved, resulting in the specialized substrates termed CELT and CELT^PLUS^. A central hypothesis of this study posits that the elevated concentration of specific cell populations within these preparations is primarily responsible for the secretion of the relevant regenerative growth factors. An overview of the regenerative impact of ADSCs on the microenvironment of a peripheral nerve injury shows Figure 1.

## 2. Materials and Methods

### 2.1. Liposuction Technique and Preparation of Lipoaspirates

The lipoaspirate used in this study was obtained from subcutaneous adipose tissue of the abdomen, thighs, and buttocks. Studies report no significant differences in the quality of lipoaspirate between these donor sites [28,29,30]. The clinical indications for the surgical liposuction were either aesthetic concerns or the treatment of lipedema. In contrast, the harvesting technique can influence the viability of the transferred cells. The lipoaspirate was collected using the wet technique, which has been described in detail before [28]. Previous studies have demonstrated that cell viability is superior with the wet technique compared to the now considered as obsolete dry technique due to its less traumatic harvesting method [29,31]. Lipoaspirates from all donors were obtained with the identical technique, by waterjet-assisted liposuction (Body-Jet^TM^, Human Med AG, Schwerin, Germany) using a tumescent solution of 0.9% NaCl with epinephrine (1:200,000). After a 15 min incubation, fat tissue was harvested with a 3.8 mm cannula under constant low negative pressure (<0.5 mbar) and transferred to the University Hospital Regensburg for further processing. The ethics review board of the University Hospital approved the study (24-3640-101).

For CELT and CELT^PLUS^ processing, the lipoaspirate was treated as previously described by Prantl et al. 2020 and Schimanski et al. 2025 [32,33]. The samples from each donor were analyzed individually. Following gravitational sedimentation, the lipoaspirate was transferred into 20 mL syringes (B. Braun Melsungen AG, Melsungen, Germany). The syringes were centrifuged at 1600 rcf for 2 min (Rotina 380R, Andreas Hettich GmbH & Co. KG, Tuttlingen, Germany), resulting in the formation of three distinct phases: an oily phase, a phase containing adipose tissue and a reddish phase consisting of tumescent solution. The oily phase and the tumescent solution phase were discarded, whereas the adipose tissue phase corresponds to CELT-processed fat. The remaining portion of the phase containing CELT fat was further processed to obtain CELT^PLUS^ fat. For this purpose, the once-centrifuged adipose tissue was transferred into 10 mL syringes (B. Braun Melsungen AG) and connected to an additional 10 mL syringe via a three-way stopcock (B. Braun Melsungen AG, Melsungen, Germany) with an inner diameter of 1.2 mm. Subsequently, the lipoaspirate was processed by standardized manual shifting (10 cycles) through a three-way stopcock. This step, during which mechanical shear forces were applied to the tissue, is referred to as intersyringe processing. A second centrifugation was then performed at 1600 rcf for 2 min. The result of the second centrifugation was again an oily phase, a reddish, presumably a cytoplasm-rich phase and a stem cell-enriched phase, corresponding to CELT^PLUS^ fat.

### 2.2. Transcriptional Analysis of Lipoaspirate Preparations: RNA-Isolation, Reverse Transcription, qPCR and Gel Electrophoresis

First, 150 mg of CELT fat was mixed with 1 mL of TRIzol (TRI Reagent^®^, Sigma Aldrich, St. Louis, MO, USA). Homogenization was performed using two syringes connected via a three-way stopcock. The resulting homogenate was centrifuged at 16,000 rcf for 5 min at 4 °C (Biofuge fresco, Heraeus, Hanau, Germany). After the first centrifugation, the red infranatant was transferred into a new 1.5 mL Eppendorf tube and centrifuged again at 12,000 rcf for 5 min at 4 °C. If a lipid layer was present on top, it was discarded, and the infranatant was mixed with 200 µL of chloroform. The mixture was shaken for 1 min and subsequently incubated at room temperature for 5 min to allow dissociation of the nucleoprotein complex. A further centrifugation step was performed at 16,000 rcf for 15 min at 4 °C. The clear supernatant was then transferred to a fresh Eppendorf tube and mixed with an equal volume of 70% ethanol. This protocol represents a slightly modified version of the method originally described by Zhang et al. (2023) [34], which was subsequently established and routinely applied within our research group [33]. The isolated RNA was purified using the RNeasy Mini Kit according to the manufacturer’s instructions (Cat no. 74104, Qiagen, Hilden, Germany). RNA concentration and quality were assessed using a spectrophotometer (NanoDrop 2000c, Thermo Fisher Scientific Inc., Waltham, MA, USA). Reverse transcription into cDNA was performed using the QuantiTect™ Reverse Transcription Kit according to the manufacturer’s instructions (Cat no. 205311, Qiagen, Hilden, Germany), which includes a genomic DNA (gDNA) removal step using gDNA wipeout buffer. The real-time PCR (Eco Real Time PCR System, Illumina, Inc., San Diego, CA, USA) was performed using the primer sequences shown in Table 1 and the Takyon™ No ROX SYBR^®^ MasterMix blue dTTP (Eurogentec, Liège, Belgium). PCR specificity was verified by agarose gel electrophoresis. Visualization was performed using a fluorescent DNA-binding dye under ultraviolet illumination (Quantum 1100, peqlab, Erlangen, Germany).

### 2.3. Translational Analysis of Lipoaspirate Preparations: Conditioning, Stimulation and ELISA

To assess the release of the key growth factors NGF and VEGF at a translational level, supernatants were prepared for enzyme-linked immunosorbent assays (ELISA).

#### 2.3.1. Conditioning

Initially, singly centrifuged CELT from one biological donor was prepared. Subsequently, 4 wells of a 6-well plate (Advanced TC Multi Plates, Greiner Bio-One GmbH, Frickenhausen, Germany) were each filled with 3 mL of the processed adipose tissue. The samples were mixed with 3 mL of Dulbecco’s Modified Eagle’s Medium (DMEM) (Sigma-Aldrich, St. Louis, MO, USA) supplemented with 1% penicillin/streptomycin (Sigma-Aldrich, St. Louis, MO, USA). DMEM served both to maintain the cells during incubation and to provide conductivity for the applied voltage during stimulation. To prevent cross-reactivity and background interference from serum-derived bovine growth factors, incubation was performed using serum-free DMEM. Tissue and medium were homogenized by gentle up-and-down pipetting using a serological pipette (Greiner Bio-One GmbH, Frickenhausen, Germany). The prepared 6-well plates were incubated at 37 °C and 5% CO_2_ (HERA Cell 240, Thermo Scientific, Waltham, MA, USA). At the end of the incubation period, the contents of each well were transferred into 10 mL syringes and centrifuged at 1600 rcf for 2 min to separate the adipose tissue from the liquid phase. For each of the four incubation time points (24, 48, 72, and 92 h), four wells were analyzed. The resulting supernatant was filtered through a 0.2 µm sterile filter (SARSTEDT AG & Co, Nümbrecht, Germany) into 1.5 mL reaction tubes.

#### 2.3.2. Stimulation

**Figure 2:**
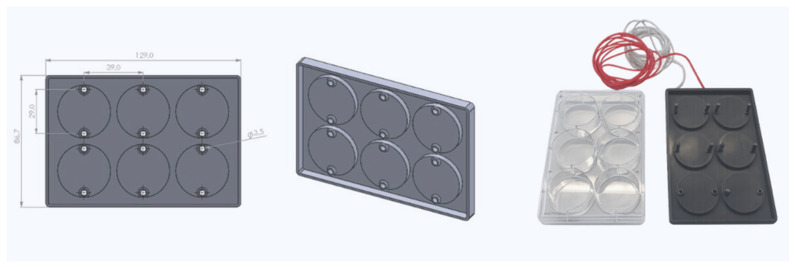
Technical design of the applied 3D-printed 6-well plate. Dimensions in the technical drawing are formatted with commas as decimal separators.

To investigate the effect of electrical stimulation on NGF secretion, a modified cover for the 6-well plate, as shown in Figure 2, was fabricated using 3D printing (Prusa Mini+ Printer, Prusa Research, Prague, Czech Republic). Graphite electrodes with a diameter of 3.5 mm were used for stimulation, with 29 mm between the cathode and anode.

Stimulation was applied under constant voltage, generating an electric field strength of 0.69–0.75 V/cm for continuous time intervals of 2, 5, 12 and 24 h. Stimulation parameters and exposure durations were selected as an exploratory approach based on previous studies investigating electrically stimulated ADSCs [35,36]. Since the present study examines processed lipoaspirate rather than isolated cell populations, a higher electrical field strength was chosen under the assumption that electrical field distribution within a heterogeneous tissue may differ from that in isolated cell culture systems. During stimulation, cells were incubated at 37 °C and 5% CO_2_, while the corresponding control samples remained unstimulated under identical environmental conditions; thereafter, both stimulated samples and unstimulated controls were incubated for an additional 48 h. Components of the culture medium served as an ion source, ensuring conductivity and thus uniform current distribution, which enabled reliable tissue stimulation. A Power Pac Basic supply (Bio-Rad Laboratories, Hercules, CA, USA) served as the current source, and the applied voltage at the electrodes of the cover was verified using a multimeter (AstroAI DM6000AR, Los Angeles, CA, USA). Initial stimulation experiments across all time points (2, 5, 12, and 24 h) and their respective controls were performed using samples from a single donor, with NGF concentrations analyzed in quadruplicate (four wells) per condition. To validate the 24 h stimulation period, this specific time point was replicated using samples from two additional independent donors, likewise analyzing four wells per treatment group.

#### 2.3.3. ELISA

Growth factor concentrations in conditioned media were measured using DuoSet ELISA kits (R&D Systems, Minneapolis, MN, USA) for human beta-NGF and human VEGF-A according to the manufacturer’s protocol. The blank samples consisted only of the reagent diluent prepared according to the manufacturer’s specifications. Samples, standards and blanks were assayed in duplicates. Optical density was measured at a wavelength of 450 nm using a Varioskan Flash microplate reader (Thermo Scientific, Waltham, MA, USA) with SkanIt Software (version 2.4.5 RE for Varioskan Flash, Thermo Scientific). The raw data were exported, and standard curve generation and calculation of growth factor concentrations were performed using Microsoft Excel (Microsoft Corporation, Redmond, WA, USA).

### 2.4. Statistical Analysis

Normally distributed data are presented as mean ± standard deviation; data deviating from normal distribution are shown as box plots with interquartile ranges.

For each growth factor, lipoaspirates from ten different donors were analyzed and ΔCt values were calculated as the difference between the cycle threshold (Ct) of the target gene and the reference gene. ΔCt values obtained under both experimental conditions (CELT and CELT^PLUS^) were assessed for normality using a Shapiro–Wilk-test. When the ΔCt values were normally distributed, a Student’s *t*-test was performed to compare the groups. For non-normally distributed data, the Wilcoxon signed-rank test was applied as a nonparametric alternative. Since individual target genes represent independent biological hypotheses regarding neurogenic and angiogenic potential, separate pairwise tests were conducted. While this exploratory approach avoids Type II errors to preserve sensitivity for distinct pathways, it carries a risk of cumulative Type I errors that should be considered when interpreting borderline significances, e.g., for *BDNF*.

For graphical representation of quantitative PCR results, normalized relative expression levels were calculated using the ΔΔCt-Method, with CELT values normalized to 1 [37,38]. For the analysis of NGF and VEGF release kinetics via ELISA, experiments were performed using technical replicates derived from a single biological donor. Specifically, four separate wells per time point were analyzed, with supernatants measured in technical duplicates. The technical duplicates of the ELISA measurements were averaged prior to inferential testing. To account for the intra-experimental variance and to correct for multiple comparisons across the consecutive time points data were analyzed using a Kruskal–Wallis test followed by a Bonferroni post hoc test for NGF and a one-way ANOVA with Bonferroni correction for VEGF, where data met parametric assumptions. To analyze the influence of electrical stimulation for different time periods on NGF kinetics, lipoaspirate from one single donor was measured in technical replicates. For each stimulation length and corresponding unstimulated control, the supernatants of 4 wells were tested. The stimulation length of 24 h was repeated with two additional lipoaspirate samples. Statistical analysis was performed via two-way ANOVA, following post hoc pairwise comparisons at each time using Bonferroni correction.

For the results of the PCRs and the ELISAs, *p*-values ≤ 0.05 were considered statistically significant (*: *p* < 0.05; **: *p* < 0.01; ***: *p* < 0.001; ns: not significant). Statistical analyses were performed using IBM SPSS Statistics (Version 20, IBM Inc., Armonk, NY, USA).

## 3. Results

The pro-regenerative secretory potential of differently processed lipoaspirate samples was evaluated by assessing the expression of growth factors essential for peripheral nerve regeneration. Analyses were performed at both the transcriptional level, using quantitative real-time PCR, and the translational level, via ELISA. Additionally, the impact of electrical stimulation on NGF expression was examined to determine its modulatory effect.

### 3.1. NGF, VEGF and BDNF Expression Is Increased, but IGF-1 Is Downregulated in CELT^PLUS^

**Figure 3:**
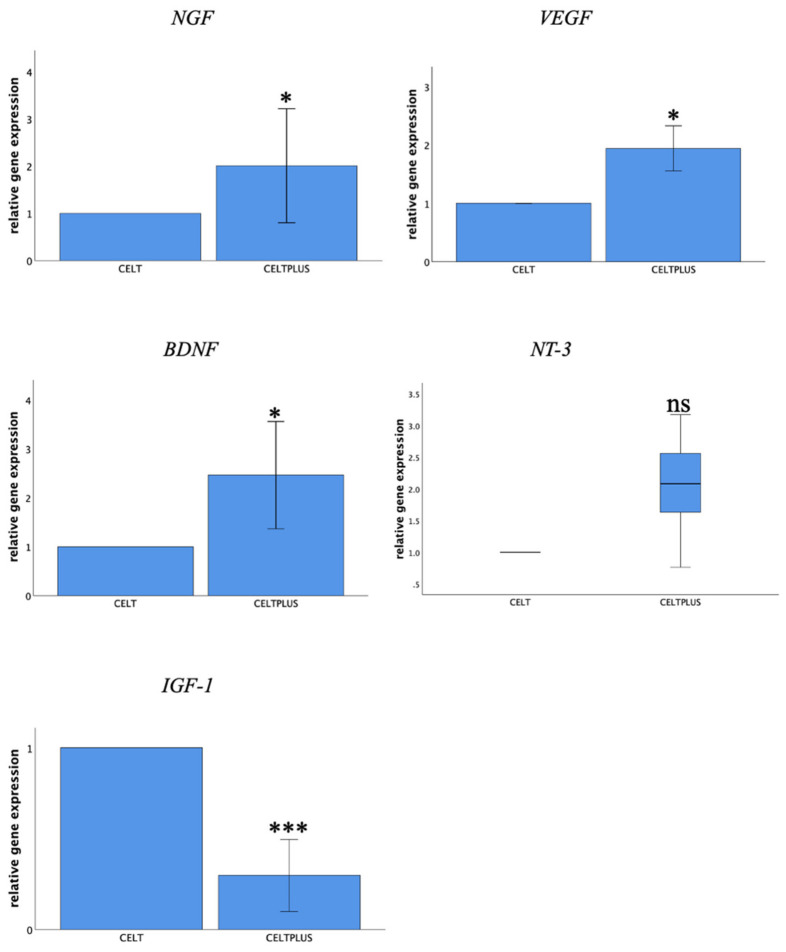
Relative gene expression of growth factors in CELT and CELT^PLUS^. For each growth factor, lipoaspirates of 10 donors have been examined. Normally distributed ΔCt values are shown as mean ± standard deviation and were analyzed using Student’s *t*-tests. As ΔCt values of *NT-3* showed a deviation from normal distribution, they were analyzed using the Wilcoxon signed-rank test and are presented as box plots with interquartile ranges. (CELT: cell-enriched lipotransfer; *NGF:* nerve growth factor; *VEGF*: vascular endothelial growth factor; *BDNF*: brain-derived neurotrophic factor; NT-3: neurotrophin-3; IGF-1: insulin-like growth factor-1; *: *p* < 0.05; ***: *p* < 0.001; ns: not significant).

Following the assessment of growth factor expression in lipoaspirate by gel electrophoresis and comparison of ΔCt values, the effect of lipoaspirate processing on growth factor expression was evaluated. Figure 3 presents the relative gene expression of the analyzed growth factors between CELT and CELT^PLUS^.

CELT^PLUS^ samples showed a significant increase in relative gene expression compared to CELT for *NGF* (*p* = 0.02), *VEGF* (*p* = 0.02), and *BDNF* (*p* = 0.04), with expression levels approximately twofold higher in CELT^PLUS^. The largest difference was observed for *BDNF*, with a relative expression of 2.47. The relative expression of *NT-3* in CELT^PLUS^ was also approximately doubled, but the increase did not reach statistical significance (*p* = 0.074). *IGF-1* was the only growth factor analyzed that exhibited a significant downregulation in CELT^PLUS^ (*p* < 0.001).

### 3.2. NGF and VEGF Production

**Figure 4:**
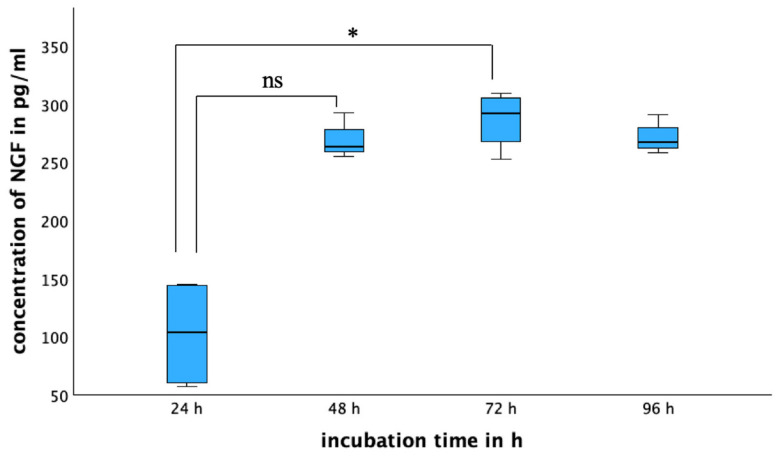
NGF concentration in lipoaspirate culture supernatants after incubation for 24, 48, 72 and 96 h. As the data deviated from normal distribution, results are shown as box blots with interquartile ranges. The figure presents data from one single biological donor. For each incubation time, data of 4 technical replicates, which have been measured in duplicates in the ELISA, are shown. The Kruskal–Wallis test with Bonferroni correction revealed a significant increase in NGF concentration between 24 and 72 h (*p* = 0.029). The comparisons of NGF concentration between the other incubation times did not reach significance. (NGF: nerve growth factor; *: *p* < 0.05; ns: not significant, h: hours).

To complement the gene expression data of the key neurogenic and angiogenic growth factors NGF and VEGF, the production of the corresponding proteins was assessed. This was achieved by measuring growth factors secreted by lipoaspirate cells directly in the conditioned media via ELISA. To determine the release kinetics of NGF and VEGF, lipoaspirate samples from one single donor were measured in 4 wells for every time point. The conditioned medium collected from each well was analyzed in duplicate using ELISA. As the measurements of NGF were not normally distributed, statistical analysis was performed using the Kruskal–Wallis test followed by Bonferroni post hoc test for multiple comparisons. Figure 4 illustrates the kinetics of NGF concentration. After 24 h, the median of NGF concentration measured 102.26 pg/mL, followed by a rapid increase over the next measurements, reaching a plateau with a maximum of 286.83 pg/mL at 72 h.

**Figure 5:**
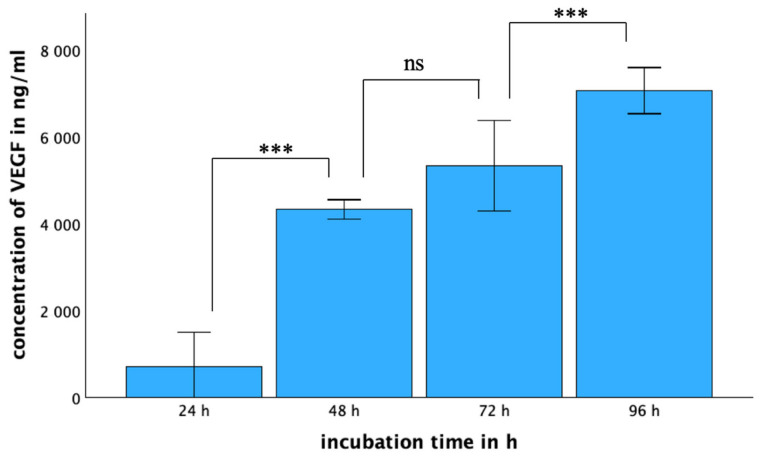
VEGF concentration in lipoaspirate supernatants after incubation for 24, 48 72 and 96 h. Data was normally distributed and is shown as mean ± standard deviation. The lipoaspirate stems from one single biological donor and was measured in technical replicates of 4 wells for every incubation time. The supernatants of each well were analyzed in duplicate in the ELISA. The one-way ANOVA with Bonferroni correction showed a significant increase between 24 and 48 h and between 72 and 96 h (both *p* < 0.001). The difference between 48 and 72 h did not reach statistical significance (*p* = 0.051). (VEGF: vascular endothelial growth factor; ***: *p* < 0.001; ns: not significant; h: hours).

VEGF release was assessed in the same time intervals. Measurements were normally distributed, and differences between time points were evaluated using a one-way ANOVA with Bonferroni correction. Figure 5 illustrates the time course of VEGF concentration, expressed in ng/mL. An increase in VEGF concentration was observed at all time points. The mean concentration rose from 697.06 ng/mL at 24 h to 7064.65 ng/mL at 96 h.

### 3.3. NGF Secretion Can Be Enhanced by Electrical Stimulation

**Figure 6:**
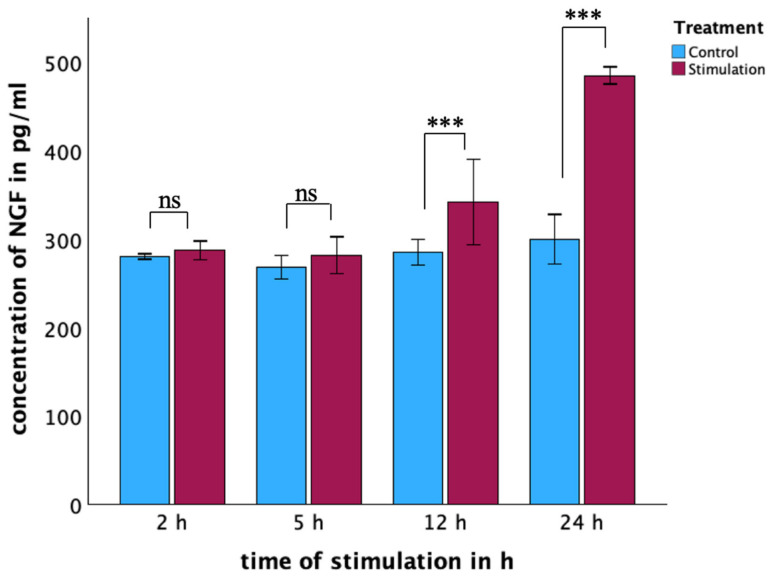
NGF concentration after Stimulation with 2 V for multiple time points. All measurements were obtained from the lipoaspirate of one single donor. The red bars represent samples that underwent electrical stimulation for the time indicated on the x-axis, whereas the controls (blue bars) were incubated for the identical time periods without stimulation. For each time point and treatment, 4 technical replicates have been analyzed in duplicates via ELISA. A two-way ANOVA followed by Bonferroni post hoc tests showed no significant differences in NGF release between stimulated samples and unstimulated controls at 2 h (*p* = 0.486) and 5 h (*p* = 0.179). Stimulation of 12 h (mean difference: 56.738 pg/mL, *p* < 0.001) and 24 h (mean difference: 184.751, *p* < 0.001) significantly increased NGF concentrations) compared to the corresponding unstimulated controls. (NGF: nerve growth factor; ***: *p* ≤ 0.001; ns: not significant; h: hours).

The effect of electrical stimulation on the secretion of the key growth factor NGF was investigated by applying a constant voltage of 2 V to CELT over different time periods. Following the stimulation period, samples were incubated for 48 h, as previous measurements indicated a marked increase in NGF concentration between 24 h and 48 h (Figure 4). A significant increase in NGF concentration compared with the unstimulated control was observed after 12 h and became more pronounced after 24 h. After 24 h of stimulation, NGF concentration was 299.52 pg/mL in the control sample and 484.27 pg/mL in the stimulated sample, demonstrating a time-dependent enhancement of NGF secretion by electrical stimulation. Measured values were normally distributed, and differences between stimulated samples and non-stimulated controls were analyzed using a two-way ANOVA with Bonferroni correction. Figure 6 illustrates the impact of stimulation duration on NGF concentrations.

**Figure 7:**
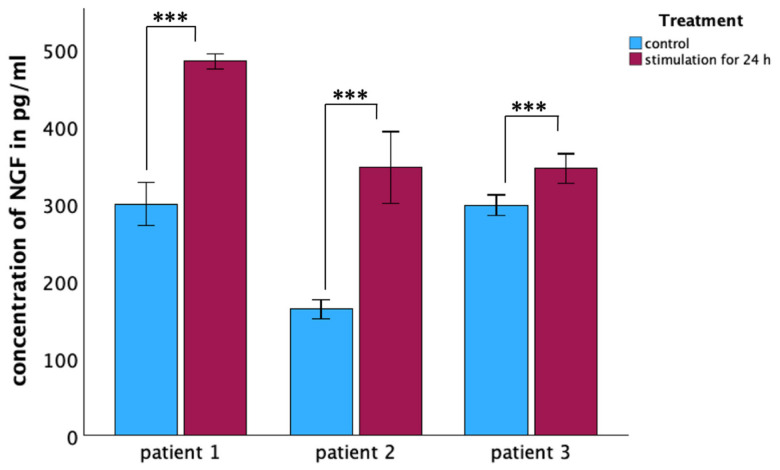
NGF concentration after stimulation with 2 V for 24 h. The impact of a 24 h stimulation on three biological replicates is shown. As in Figure 6, the red bars represent stimulated samples and the blue bars unstimulated controls. For each patient and treatment (control vs. stimulation for 24 h) supernatants of 4 wells have been analyzed in duplicates via ELISA. The data of patient 1 is already shown in Figure 6. Two-way ANOVA and Bonferroni-corrected post hoc analysis demonstrated that the stimulation led to a significant increase in NGF concentration in the two additional patients compared to their unstimulated controls (mean difference of patient 2: 183.065 pg/mL, mean difference of patient 3: 47.069 pg/mL, all *p* < 0.001). (NGF: nerve growth factor; ***: *p* ≤ 0.001; h: hours).

To validate the inductive effect observed after 24 h of stimulation, this experimental setup was repeated under identical parameters using lipoaspirate from two additional independent donors (patient 2 and patient 3, Figure 7). For a comprehensive comparison, the corresponding 24 h stimulation data from the first donor (patient 1, originally shown in Figure 6) were integrated into Figure 7 alongside the new donors.

As illustrated in Figure 7, electrical stimulation resulted in increased NGF concentrations in all analyzed samples compared with their respective controls. The observed differences were statistically significant in all three patients, confirming a stimulatory effect of the treatment on NGF protein secretion.

## 4. Discussion

The ability of ADSCs and cells of the SVF to form neurotropic, neurotrophic and angiogenic growth factors is described in the current literature [39,40,41]. These cell populations generate a broad spectrum of paracrine signals that act as crucial mediators during nerve and tissue regeneration. The intersyringe processing applied to produce CELT^PLUS^ specifically alters the composition of the lipoaspirate. Larger, lipid-containing adipocytes, whose regenerative potential is considered limited, are mechanically disrupted and removed, while cell fractions that contribute to regeneration are simultaneously enriched [28]. Previous studies have demonstrated through histological analyses and quantitative cell characterization that the CELT^PLUS^ processing protocol results in reproducible enrichment of ADSC- and SVF-associated cellular populations [22,32]. Collectively, these findings support the hypothesis that CELT^PLUS^ preparations contain an increased proportion of regenerative cell populations, potentially accounting for the enhanced secretion of regeneration-associated growth factors. This interpretation is in line with the gene expression patterns observed in the present study. Our data of the relative gene expression indicated a significant two-fold increase for *NGF*, *BDNF* and *VEGF*, and for *NT-3* an enhanced, although not significant, upregulation in CELT^PLUS^. These results suggest that CELT^PLUS^ is associated with an enhanced expression profile of neurogenic and angiogenic growth factors. *BDNF* is a well-established neurotrophic factor that supports neuronal survival and axonal outgrowth, with a particularly important role during the later phases of peripheral nerve regeneration [10]. Given the pivotal roles of *NGF* and *VEGF* during the earlier phases of regeneration, the present study focused on the secretion of these key regenerative growth factors, whereas *BDNF* was assessed only at the transcriptional level. Nevertheless, the observed changes in *BDNF* gene expression provide a promising foundation for future studies investigating the relationship between transcriptional regulation and protein secretion. The PCR results showed that only *IGF-1* expression was significantly reduced in the CELT^PLUS^ samples. This observed reduction may be related to the altered cellular composition of the lipoaspirate induced by intersyringe processing. Together with the indicated increased expression of several neurogenic and angiogenic growth factors, these findings suggest that CELT^PLUS^ processing is associated with a shift in the molecular profile of the lipoaspirate toward a more pro-regenerative growth factor expression pattern. As highlighted in Section 1, *IGF-1* is among the key mediators of peripheral nerve regeneration. Given the observation that *IGF-1* expression was significantly lower in CELT^PLUS^, the supplementation of additional *IGF-1* via an alternative medium could be a possible target to enhance nerve regeneration. A potential option would be the additional application of platelet-rich plasma (PRP), which is known to contain IGF-1 alongside many other growth factors [42,43]. In fact, targeted supplementation with PRP may represent a clinically feasible strategy to augment endogenous IGF-1 levels without substantially increasing the injection volume at the lesion site. *GAPDH* was selected as a housekeeping gene based on its established application in human white adipose tissue even under strong external stimuli and stability even after differentiation [44]. To standardize the experimental setup, RNA input was strictly quantified and equalized prior to cDNA synthesis. However, the use of *GAPDH* as the sole reference gene for relative quantification represents a methodological limitation in the interpretation of the qPCR data in the present work.

The pro-regenerative expression pattern of CELT was further investigated at the translational level. This allows for an assessment of the dynamics of protein secretion for NGF and VEGF by ADSCs and cells of the SVF. The concentration of these factors was measured in the conditioned supernatants at four different time intervals. For NGF, rapid secretion was observed within the first 48 h, which subsequently transitioned into a plateau. The concentration of VEGF increased continuously across all measurement time points.

The strong increase in NGF concentration within the first 48 h with subsequent stagnation can be interpreted as an indication of an early, limited release phase. The cause could be a pronounced initial activation of the cells or an early exhaustion of intracellular NGF stores. In contrast, the continuous increase in VEGF points toward sustained transcription and secretory activity. A possible cause for the dynamics of NGF release could be a stronger stress-induced secretion compared to VEGF, which causes the initial increase. The early NGF plateau, on the other hand, could be an expression of exhaustion of secretory activity or a downregulation of the corresponding signaling pathways. The increase in VEGF concentration could also indicate an adaptation of the cells to ex vivo conditions, such as hypoxia or a decreasing nutrient gradient, which enhance the release of VEGF as an angiogenic response. Another reason for the differences could be the activity of the involved cell populations of the lipoaspirate. Early NGF secretion could primarily originate from cell populations whose activity decreases over time, while VEGF is increasingly formed by stromal and endothelial progenitor cells whose activity is maintained over a longer period. Different biological functions and context-dependent expression could also play a role. NGF acts primarily neurotrophically and immunomodulatorily [45,46], functions that are particularly relevant in early stress and repair phases. As described initially, NGF contributes to a transition from the initial inflammatory response to tissue regeneration. VEGF mediates angiogenic adaptation processes that can remain active over several days [47]. VEGF-mediated vascularization is critical during the early stages of nerve injury, including the inflammatory response and Wallerian degeneration, where neovascularization enables the recruitment of repair-associated cells and the clearance of cellular debris at the lesion site. Moreover, it ensures adequate metabolic and trophic support during the subsequent phases of Büngner band formation and axonal reinnervation [15,47].

The release dynamics of growth factors from lipoaspirate may therefore reflect the varying roles of the factors during different phases of regeneration. The substantially higher VEGF concentrations observed indicate a more pronounced secretory activity for this factor. This secretion pattern is consistent with the concept that NGF may primarily contribute to the initial activation of the regenerative microenvironment following PNI and subsequently exert a more modulatory role, whereas VEGF mainly supports regeneration over time by promoting vascularization through angiogenesis. One limitation in interpreting the secretion dynamics lies in the use of processed lipoaspirate instead of a monoculture of ADSCs. Since the exact cell count per population, potential cell division processes and cell viability in native tissue cannot be precisely quantified, the exact dynamics of growth factor secretion cannot be standardized to a defined cell count. Variations in cellular composition between lipoaspirate samples may have contributed to the observed differences in protein secretion profiles. Although the number of donors included in the protein secretion experiments was limited, previous studies by Eigenberger et al. [22] demonstrated consistent compositional changes during the processing of CELT and CELT^PLUS^ preparations across more than 40 histological samples from multiple donors, including a reproducible increase in stromal vascular cell density. These findings support the robustness and reproducibility of the processing protocol. Nevertheless, individual donor-specific differences in cellular composition and secretory activity may still contribute to biological variability. Future studies including larger donor cohorts will further refine our understanding of inter-individual variability and strengthen the characterization of the secretory properties of CELT-derived preparations. However, the key finding of this study is the demonstration of growth factor secretion and dynamics directly from the tissue, as this would enable effective clinical application.

To employ a transplant with an even higher pro-regenerative expression pattern, an attempt was made to increase the concentration of NGF through electrical stimulation of the lipoaspirate. Following stimulation and subsequent incubation, a significant increase in the growth factor was detected in three supernatants.

Previous studies by several research groups, including Zocchi et al. [40] and Lavorato et al. [48], have shown that hypoxia and electrical stimulation enhance the secretion of regeneration-associated growth factors by ADSCs relevant for PNI. Along similar lines, Hlavac et al. [35] electrically stimulated commercially available human ADSCs for 24 h at a frequency of 1 Hz for 24 h. After 24 h of incubation, a significant increase in the factors VEGF and BDNF was detected in the supernatant compared to the control. Subsequently, a neurite outgrowth assay was performed with this supernatant, showing significant neurite elongation of cells from the SY5Y cell line, comparable to a positive control medium. A publication by Beugels et al. [36] showed that electrical stimulation significantly increased the secretion of pro-angiogenic factors, particularly VEGF. Despite the increase in proteins such as TSP-1 with opposing effects, a CAM assay showed a clearly dominant pro-angiogenic effect. A further positive outlook for the clinical application of processed lipoaspirate is presented by the work of Schimanski et al. [33]. It proves that even after cryopreservation of CELT and CELT^PLUS^, a portion of the cells is damaged; however, cells of the SVF retain high vitality and functionality. The possibility of preservation would allow for long-term treatment after a single liposuction.

Although cell viability was not assessed in this study, preliminary experiments, together with the available evidence, have demonstrated that the applied voltages do not adversely affect cell viability in ADSCs [36]. Still, potential effects of electrical stimulation on cell viability in this experiment cannot be fully excluded.

Although serum-free conditions were necessary to avoid contamination of the secretome with bovine growth factors, prolonged serum deprivation may have induced cellular stress, potentially influencing growth factor release kinetics. In addition, the protein expression analyses were performed using samples from a single donor, and therefore do not capture potential inter-individual variability. While this limits the generalizability of the quantitative secretion kinetics, the study provides an important proof of concept and, to our knowledge, one of the first characterizations of the immediate growth factor secretion profile of freshly harvested lipoaspirate in response to electrical stimulation. These findings establish a valuable foundation for future studies including larger donor cohorts, which will enable validation of the observed secretion patterns and further characterize donor-dependent biological variability.

In the present work, intersyringe processing increased without enzymatic digestion the relative density of SVF-associated cell populations like ADSCs. This was accompanied by an approximately two-fold higher gene expression observed for *NGF*, *BDNF*, *VEGF*, and *NT-3*. The experiments on the dependence of incubation time on the concentration of NGF and VEGF allow for conclusions regarding the secretion dynamics of the cell populations and served as an indication for the settings of the stimulation experiment, which showed that the secretion of NGF can be increased by electrical stimulus. In connection with the previously cited publications, the hypothesis is strengthened that lipoaspirate preparations through tissue engineering could be an effective transplant for improved regeneration after PNI. The potential could be further improved, for example, by stimulating cell populations in CELT^PLUS^. The stimulation experiments with subsequent NGF determination showed a significant increase in all three patients.

## 5. Conclusions

The results of this study have demonstrated that the mechanical processing of autologous adipose tissue induces a modulation of both the secretory and transcriptional profiles of key growth factors. Gene expression analyses indicate higher *mRNA* levels of various neurogenic and angiogenic factors in CELT^PLUS^. Furthermore, proteomic analysis of the conditioned media confirms a time-dependent release of these factors, validating the functional activity of the resident ADSCs and the SVF. Additionally, the application of electrical stimulation further enhanced NGF secretion, highlighting the susceptibility of the secretory potential of adipogenic cell populations to external modulation. The present findings are promising but are limited because of the biological replication and analyses on transcriptional and protein levels. As a next step, functional in vitro assays, such as neurite outgrowth assays proving axonal sprouting or CAM assays evaluating angiogenesis, should validate whether these molecular changes result in enhanced neural regeneration.

## Figures and Tables

**Table 1:** Primer sequences and specifications used for real-time RT-qPCR analysis. Abbreviations and NCBI gene IDs (NGF: nerve growth factor, ID: 4803; VEGF: vascular endothelial growth factor, ID: 7422; BDNF: brain-derived neurotrophic factor, ID: 627; NT-3: neurotrophin-3, ID: 4908; IGF-1: insulin-like growth factor 1, ID: 3475; GAPDH: glyceraldehyde-3-phosphate dehydrogenase, ID: 2597). qPCR efficiency was assumed to be approximately 2 (100%) based on pre-optimization via Primer-BLAST (NCBI, Bethesda, MD, USA; Primer3 v 2.5.0) and confirmation of strict specificity via post-amplification melting curve analysis.

Gene	Forward and Reverse Primer	Melting Temperature in °C
NGF	5′-CACACTGAGGTGCATAGCGT-3′ 5′-AGTGTGGTTCCGCCTGTATG-3′	62 °C
VEGF	5′-AACCAGCAGAAAGAGGAAAGAGG-3′ 5′-CCAAAAGCAGGTCACTCACTTTG-3′	60 °C
BDNF	5′-CTGGAGCCAGAATCGGAAC-3′ 5′-GAACTCGGGGTCCACACAAA-3′	65–55 °C (Touchdown)
NT-3	5′-CCGTGGCATCCAAGGTAACAA-3′ 5′-GCAGTTCGGTGTCCATTGC-3′	65–55 °C (Touchdown)
IGF-1	5′-GCTCTTCAGTTCGTGTGTGGA-3′ 5′-GCCTCCTTAGATCACAGCTCC-3′	60 °C
GAPDH	5′-GGGAGCGAGATCCCTCCAAAAT-3′ 5′-GGCTGTTGTCATACTTCTCATGG-3′	60 °C

## Data Availability

The original contributions presented in this study are included in the article. Further inquiries can be directed to the corresponding author.

## Abbreviations

ADSC	Adipose-derived stem cell
BDNF	Brain-derived neurotrophic factor
Bp	Base pair
CAM assay	Chorio-allantoic-membrane assay
cDNA	Complementary DNA
CELT	Cell-enriched lipotransfer
cm	centimeter
CO_2_	Carbon dioxide
Ct	Cycle treshold
DMEM	Dulbecco’s modified eagle’s medium
DNA	Desoxyribonucleic acid
ELISA	Enzyme-linked immunosorbent assay
GAPDH	Glyceraldehyde 3-phosphate dehydrogenase
GDNF	Glial cell-derived neurotrophic factor
IGF-1	Insulin-like growth factor 1
mbar	milibar
NaCl	Sodium chloride
NCBI	National center for biotechnology information
ng	nanogram
NGC	Nerve guide conduit
NGF	Nerve growth factor
ns	not significant
NT-3	Neurotrophin-3
*p*	*p*-value
PBS	Phosphate-buffered saline
pg	picogram
PNI	Peripheral nerve injury
PRP	Platelet-rich plasma
qPCR	Quantitative polymerase chain reaction
Rcf	Relative centrifugal force
RNA	Ribonucleic acid
SC	Schwann cell
SVF	Stromal vascular fraction
UV	Ultraviolet
V	Volt
VEGF	Vascular endothelial grwoth factor
